# Effect of Hyperinsulinemia and Insulin Resistance on Endocrine, Metabolic, and Reproductive Outcomes in Non-PCOS Women Undergoing Assisted Reproduction: A Retrospective Cohort Study

**DOI:** 10.3389/fmed.2021.736320

**Published:** 2022-01-07

**Authors:** Wang-Yu Cai, Xi Luo, Jianyuan Song, Danpin Ji, Jun Zhu, Cuicui Duan, Wei Wu, Xiao-Ke Wu, Jian Xu

**Affiliations:** ^1^Fourth Affiliated Hospital, Zhejiang University School of Medicine, Yiwu, China; ^2^Department of Obstetrics and Gynecology, The Second Affiliated Hospital of Zhejiang Chinese Medical University, Hangzhou, China; ^3^Zhejiang University School of Medicine, Hangzhou, China; ^4^Women's Hospital, Zhejiang University School of Medicine, Hangzhou, China; ^5^Department of Gynecology, First Affiliated Hospital, Heilongjiang University of Chinese Medicine, Harbin, China; ^6^Heilongjiang Province Hospital, Harbin, China

**Keywords:** assisted reproduction, insulin resistance, hyperinsulinemia, metabolic, ovarian stimulation

## Abstract

**Objective:** To evaluate the effect of hyperinsulinemia (HI) and insulin resistance (IR) on endocrine, metabolic, and reproductive outcomes in women without polycystic ovary syndrome (PCOS) undergoing assisted reproduction.

**Materials and Methods:** The study included 1,104 non-PCOS women undergoing *in vitro* fertilization/intracytoplasmic sperm injection-fresh embryo transfer. HI was evaluated by serum fasting insulin (FIN), and IR was evaluated by homeostatic model assessment of insulin resistance index (HOMA-IR). In addition, biometric, sex hormone, and metabolic parameters were measured. Independent *t*-test, linear, and logistic regression examined associations between HI, IR, and endocrine, metabolic, ovarian stimulation characteristics, and reproductive outcomes.

**Results:** Women with HI and IR had lower levels of progesterone, luteinizing hormone, follicle-stimulating hormone, estradiol, high-density lipoproteins, and increased levels of triglycerides low-density lipoproteins. For ovarian stimulation characteristics, those with HI and IR had a longer duration of stimulation, a higher total gonadotropin dose, and a lower peak estradiol level. Linear regression confirmed these associations. For reproductive outcomes, HI and IR were not associated with clinical pregnancy, live birth, and miscarriage.

**Conclusions:** HI and IR did not impair reproductive outcomes in non-PCOS women undergoing assisted reproduction.

## Introduction

Infertility and assisted reproduction technology (ART) are associated with considerable emotional and financial burdens. However, it is usually difficult to predict the results of assisted reproduction because it is influenced by multiple factors such as the social, psychological, and physical status of the patient (1–3).

Hyperinsulinemia (HI) and insulin resistance (IR) status have been well-documented to be associated with an increased risk of type 2 diabetes, hypertension, cardiovascular disease, and non-alcoholic fatty liver disease (4–6). Insulin has also been suggested to play an important role in female reproductive health and follicle development. Periovulatory insulin signaling is essential for ovulation, cell differentiation of the granulosa, and female fertility (7). An *in vitro* study found that IR could decrease steroidogenesis and increase apoptosis of human granulosa cells (8). Preimplantation exposure to high concentrations of insulin-like growth factor I results in lower implantation rates in mice (9). Elevated insulin levels could compromise mice decidualization in early-stage pregnancy (10). Previous studies suggested that IR was an independent risk factor for spontaneous abortion and recurrent pregnancy loss (11, 12).

IR and HI are found more frequently in women with polycystic ovary syndrome (PCOS) (13, 14). HI and IR can lead to worse endocrine, metabolic, and fertility outcomes in women with PCOS who underwent ovulation induction (15). Preconception impaired glucose tolerance was independently associated with adverse pregnancy outcomes in women with PCOS who underwent fresh or frozen embryo transfer (16). A systematic review found that IR is a risk factor for spontaneous abortion in women with PCOS who underwent assisted reproductive treatment (17).

Despite all of the above, the evidence for the effect of HI and IR on women without PCOS is limited. Therefore, we designed this study to investigate associations between HI, IR, and endocrine, metabolic, and reproductive outcomes in a cohort of women who did not have PCOS.

## Materials and Methods

### Study Design

This was a retrospective cohort study of subjects from January 2018 to October 2020 in a center for reproductive medicine of Women's Hospital, Zhejiang University School of Medicine in China. The study was approved by the ethics committee of the hospital (IRB-20200235-R) and was conducted in accordance with the Declaration of Helsinki. Because this was a retrospective study and only de-identified data were analyzed, the ethics committee waived the standard requirement of informed consent from the women in the study.

### Study Population

**Figure 1:**
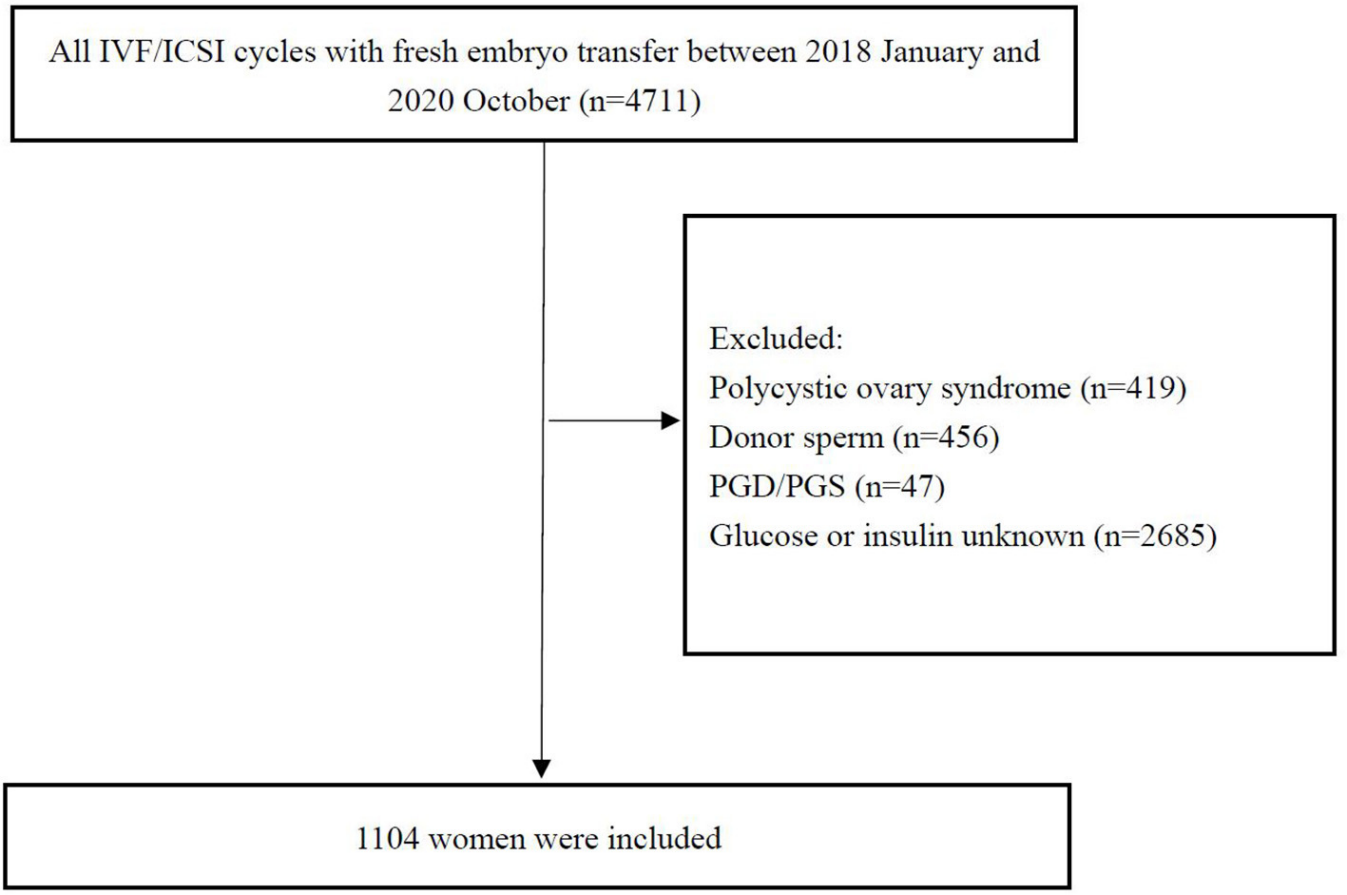
Flowchart for selecting women included in this study.

Patients undergoing the first *in vitro* fertilization (IVF)/intracytoplasmic sperm injection (ICSI) cycle with fresh embryo transfer from January 2018 to October 2020 were recruited in the study. Briefly, the inclusion criteria were the following: (1) women 18–45 years old; (2) fresh embryo transfer and IVF/ICSI; and (3) fasting insulin (FIN) and glucose (FG) evaluated before treatment. The exclusion criteria were (1) women with the diagnosis of PCOS: PCOS was diagnosed by Rotterdam criteria [two out of three of oligo-amenorrhea, biochemical or clinical hyperandrogenism, and polycystic ovaries on transvaginal ultrasound (18)]; (2) the use of an egg or sperm donor; (3) preimplantation genetic diagnosis or screening; (4) lack of FIN and glucose evaluation. Data from 4,711 women were initially screened, of which 1,104 were included in the final analysis. A flow chart of the study procedure is shown in Figure 1.

At the initial evaluation, age, height, weight, systolic blood pressure (SBP), and diastolic blood pressure (DBP) were measured, and body mass index (BMI) was calculated. Reproductive data included infertility diagnosis, primary infertility, and duration of infertility. Blood samples were collected after an overnight fast on Day 2–4 of the menstrual cycle prior to the beginning of pituitary down-regulation. Anti-Mullerian hormone (AMH), basal follicle-stimulating hormone (FSH), basal luteinizing hormone (LH), basal estradiol (E2), basal progesterone (P), basal testosterone (T), basal prolactin (PRL), total cholesterol (TC), low-density lipoprotein (LDL), high-density lipoprotein (HDL), triglycerides (TG), FG, and FIN were measured. Insulin resistance assessment of the homeostasis model (HOMA-IR) was calculated using the formula [HOMA-IR = (FIN × FG)/22.5]. HI was defined as the upper 10 percentile of FIN in this current cohort. IR was defined as HOMA-IR >2.69 according to a previous study (19).

### Laboratory Assessment

FSH, LH, E2, T, and PRL were measured by chemiluminescence using a Roche E801 analyzer (Roche, Switzerland) according to the manufacturer's protocol. TC, HDL, LDL, and TG were measured using a Beckman AU5800 chemistry analyzer (Beckman Coulter, United States) according to the manufacturer's protocol. FG was measured using a Beckman AU5800 chemistry analyzer (Beckman Coulter, United States). The intra-assay laboratory coefficients of variation for FG ranged from 0.97 to 1.03%, and the between-assay coefficients of variation ranged from 1.40 to 2.96%. Finally, FIN was measured using an Abbott i2000SR immunoassay analyzer (Abbott, United States). The intra-assay laboratory coefficients of variation for FIN ranged from 1.75 to 1.80%, and the between-assay coefficients of variation ranged from 1.14 to 1.45%.

### Intervention

In the short-acting gonadotrophin-releasing hormone agonist protocol (GnRH-a) (Triptorelin, Ferring AG, Germany), GnRH-a was administered daily in the mid-luteal phase until trigger. After 14 days, serum LH, FSH, and E2 were measured, and recombinant FSH (Gonal-F, Merck Serono, Switzerland) was administered daily when FSH and LH were < 5 IU/L and E2 was < 50 pg/mL. In the gonadotrophin-releasing hormone antagonist (GnRH-A) (Cetrorelix, Merck Serono, France) protocol, recombinant FSH was administered on Day 2 or 3 of the menstrual cycle until the trigger, and GnRH-A was used daily when the leading follicles reached a mean diameter of 14 mm until the trigger. In both protocols, when three follicles reached a mean diameter of 17 mm, or two follicles reached a mean diameter of 18 mm, recombinant hCG (Ovidrel, Serono, Italy) was administered subcutaneously. Oocyte retrieval was performed 36 h after recombinant hCG injection. The retrieved oocytes were inseminated by conventional IVF, ICSI, or IVF/ICSI (50% IVF and 50% ICSI). The embryos were cultured in G1-plus medium (Vitrolife, Switzerland) at 37°C in an incubator with 6% carbon dioxide. Up to 2 embryos were transferred at the cleavage stage on Day 3 after fertilization.

### Outcome Measures

The outcomes of ovarian stimulation were the duration of ovarian stimulation, the total dose of gonadotropin, the peak E2, the endometrial thickness, the number of retrieved oocytes, the number of two-pronuclear embryos (2PN), and the number of embryo transfers. Reproductive outcomes were live birth, clinical pregnancy, and miscarriage. Live birth was defined as the delivery of a viable infant after 28 weeks of gestation. Clinical pregnancy was confirmed by visualization of at least one gestational sac on ultrasound. Miscarriage was defined as a pregnancy loss before 28 weeks of gestation (20).

### Statistical Analysis

SPSS statistics 24.0 (IBM) was used to analyze the data. Means and standard deviations, frequencies, and percentages derived from demographic and reproductive characteristics data were calculated. The independent *t*-test was used to compare the means between groups. Linear regression was used to evaluate associations between FIN, HOMA-IR, and outcomes of endocrine, metabolic, and ovarian stimulation before and after adjustment for age and BMI. Effects were described as coefficients β with a 95% confidence interval (CI). Logistic regression analyses were used to identify potential effects of FIN and HOMA-IR on clinical pregnancy, live birth, and miscarriage before and after adjusting for age and BMI. The effects were described as odds ratios (OR) with 95% CI. All reported *p-values* are two-sided, and *p* < 0.05 was considered statistically significant.

## Results

### Baseline Characteristics

The baseline characteristics of the 1,104 women included in the study are shown in Table 1. The mean age was 31.2 ± 4.2 years, and the mean BMI was 21.7 ± 2.8. Infertility diagnosis, duration of infertility, primary infertility, AMH, basal E2, LH, FSH, P, T, PRL, TC, LDL, HDL, TG, glucose, FIN, and HOMA-IR were also reported in Table 1. The mean FIN and HOMA-IR were 7.5 ± 3.6 μU/ml and 1.7 ± 0.9. HI and IR were identified in 108 (9.8%) and 133 (12.0%) women. In addition, the duration of ovarian stimulation, the total dose of gonadotropin, the peak level of E2, the thickness of the endometrial, the type of fertilization, the number of retrieved oocytes, the number of 2PN, and the number of embryo transfers were listed in Table 2. A total of 442 women (40.0%) achieved clinical pregnancy, 290 (26.2%) had live births, and 152 (34.4%) had miscarriages.

**Table 1:** Characteristics of included women.

	**Mean ± SD or number (%)**
Age (year)	31.2 ± 4.2
Height (cm)	159.9 ± 8.3
Weight (kg)	55.7 ± 7.8
BMI (kg/m^2^)	21.7 ± 2.8
SBP (mmHg)	114.7 ± 10.5
DBP (mmHg)	71.4 ± 8.6
Infertility diagnosis (*n*, %)
Female	612 (55.4)
Male	209 (18.9)
Unexplained	99 (9.0)
Other/mixed	184 (16.7)
Primary infertility (%)	539 (48.8)
Duration of infertility (year)	3.2 ± 2.8
AMH (ng/ml)	3.1 ± 1.9
Basal E2 (pg/mL)	32.8 ± 17.2
Basal FSH (IU/L)	6.7 ± 2.6
Basal LH (IU/L)	5.1 ± 2.6
Basal progesterone (nmol/L)	1.1 ± 0.7
Basal testosterone (nmol/L)	0.6 ± 0.6
Basal prolactin (ng/ml)	14.5 ± 12.3
FG (mmol/L)	5.1 ± 0.5
FIN (μU/ml)	7.5 ± 3.6
HOMA-IR	1.7 ± 0.9
HDL (mmol/L)	1.3 ± 0.3
LDL (mmol/L)	2.6 ± 0.7
TC (mmol/L)	4.4 ± 0.8
TG (mmol/L)	1.1 ± 0.6

*Data are presented as mean ± sd or number (%). BMI, body mass index; SBP, systolic blood pressure; DBP, diastolic blood pressure; AMH, anti-Mullerian hormone; E2, estradiol; FSH, follicle-stimulating hormone; LH, luteinizing hormone; FG, fasting glucose; FIN, fasting insulin; HOMA-IR, homeostatic model assessment-insulin resistance; HDL, high-density lipoprotein; LDL, low-density lipoprotein; TC, total cholesterol; TG, triglycerides*.

**Table 2:** Treatment outcomes of included women.

	**Mean ± SD or number (%)**
Duration of stimulation (day)	10.1 ± 2.2
Total gonadotropin dose (IU)	2,194.7 ± 858.7
Fertilization procedure (*n*, %)
IVF	764 (69.2)
ICSI	292 (26.4)
IVF + ICSI	48 (4.3)
Peak E2 (pg/ml)	2,457.0 ± 1,205.2
Endometrial thickness (mm)	10.8 ± 2.4
Number of oocytes retrieved	9.4 ± 4.4
Number of 2PN	5.0 ± 3.2
Number of embryo transfers	1.8 ± 0.4
Clinical pregnancy^a^ (*n*, %)	442 (40.0)
Live birth^a^ (*n*, %)	290 (26.2)
Miscarriage^b^ (*n*, %)	152 (34.4)

*Data are presented as mean ± sd or number (%)*.

a *Clinical pregnancy and live birth rates were calculated among all women*.

b *Miscarriage rate was calculated among pregnant women. IVF, in vitro fertilization; ICSI, intracytoplasmic sperm injection; E2, estradiol; 2PN, two-pronuclear embryo*.

Baseline clinical and biochemical characteristics based on the presence or absence of HI and IR are presented in Table 3. Women with HI had significantly lower P, LH, FSH, and E2 levels than women without HI. Similarly, IR was associated with lower levels of P, LH, FSH, and E2. AMH and PRL were not associated with HI and IR (Table 3).

**Table 3:** Characteristics of women with hyperinsulinemia and insulin resistance.

	**Hyperinsulinemia**			**Insulin resistance**		
	**With *N* = 108**	**Without *N* = 996**	***P*-value**	**With *N* = 133**	**Without *N* = 971**	***P*-value**
Age (year)	31.1 ± 3.9	31.2 ± 4.2	0.777	31.2 ± 4.0	31.2 ± 4.2	0.957
Height (cm)	159.3 ± 16.1	159.9 ± 7.0	0.956	159.6 ± 14.7	159.9 ± 7.1	0.561
Weight (kg)	55.7 ± 8.4	55.8 ± 7.7	0.471	56.1 ± 8.1	55.7 ± 7.8	0.760
BMI (kg/m2)	21.6 ± 3.3	21.7 ± 2.8	0.518	21.7 ± 3.1	21.7 ± 2.8	0.921
Duration of infertility	3.3 ± 2.6	3.2 ± 2.8	0.666	3.3 ± 2.7	3.2 ± 2.8	0.575
AMH (ng/ml)	3.0 ± 2.0	3.1 ± 1.9	0.615	3.0 ± 1.9	3.1 ± 1.9	0.839
Progesterone (nmo/L)	**1.0** **±** **0.8**	**1.1** **±** **0.7**	**0.036**	**1.0** **±** **0.8**	**1.1** **±** **0.7**	**0.013**
Testosterone (nmol/L)	0.7 ± 0.6	0.6 ± 0.6	0.219	0.7± 0.6	0.6 ± 0.6	0.208
LH (IU/L)	**4.4** **±** **2.4**	**5.1** **±** **2.6**	**0.006**	**4.5** **±** **2.7**	**5.1** **±** **2.5**	**0.006**
FSH (IU/L)	**5.8** **±** **2.0**	**6.8** **±** **2.7**	**0.000**	**5.9** **±** **1.9**	**6.8** **±** **2.7**	**0.000**
E2 (pg/ml)	**28.4** **±** **18.5**	**33.3** **±** **17.0**	**0.005**	**29.2** **±** **17.3**	**33.3** **±** **17.2**	**0.009**
Prolactin (ng/ml)	13.6 ± 10.9	14.7 ± 12.4	0.382	14.3 ± 13.3	14.6 ± 12.2	0.786
SBP (mmHg)	115.3 ± 10.1	114.6 ± 10.5	0.542	114.8 ± 10.4	114.7 ± 10.5	0.889
DBP (mmHg)	72.2 ± 7.5	71.3 ± 8.7	0.324	72.2 ± 7.4	71.3 ± 8.8	0.248
FG (mmol/L)	**5.3** **±** **0.4**	**5.1** **±** **0.5**	**0.000**	**5.5** **±** **0.8**	**5.1** **±** **0.4**	**0.000**
FIN (μU/ml)	**15.4** **±** **4.0**	**11.9** **±** **2.3**	**0.000**	**14.6** **±** **4.1**	**6.5** **±** **2.2**	**0.000**
HOMA-IR	**3.6** **±** **1.0**	**1.5** **±** **0.6**	**0.000**	**3.5** **±** **0.9**	**1.5** **±** **0.5**	**0.000**
HDL (mmol/L)	**1.2** **±** **0.2**	**1.4** **±** **0.3**	**0.000**	**1.2** **±** **0.2**	**1.4** **±** **0.3**	**0.000**
LDL (mmol/L)	2.7 ± 0.7	2.6 ± 0.7	0.408	**2.7** **±** **0.7**	**2.6** **±** **0.7**	**0.034**
TC (mmol/L)	4.4 ± 0.8	4.4 ± 0.8	0.744	4.5 ± 0.8	4.4 ± 0.8	0.443
TG (mmol/L)	**1.4** **±** **0.7**	**1.0** **±** **0.5**	**0.000**	**1.4** **±** **0.7**	**1.0** **±** **0.5**	**0.000**

*BMI, body mass index; AMH, anti-Mullerian hormone; LH, luteinizing hormone; FSH, follicle-stimulating hormone; E2, estradiol; SBP, systolic blood pressure; DBP, diastolic blood pressure; FG, fasting glucose; FIN, fasting insulin; HOMA-IR, homeostatic model assessment-insulin resistance; HDL, high-density lipoprotein; LDL, low-density lipoprotein; TC, total cholesterol; TG, triglycerides. Bold values indicate P-value < 0.05*.

The results also demonstrated that women with HI had significantly higher levels of FG and TG and lower HDL levels. Similarly, women with IR had significantly higher levels of FG, LDL, and TG and lower HDL levels. Blood pressure and TC did not have an association with HI and IR (Table 3).

### Characteristics of Ovarian Stimulation and Reproductive Outcomes

The results in Table 4 demonstrated that women with HI had a significantly longer duration of stimulation and higher total gonadotropin dose but a lower peak level of E2. Similarly, women with IR had a significantly longer duration of stimulation and higher total gonadotropin dose but a lower peak E2. Endometrial thickness, number of retrieved oocytes, number of 2PN, clinical pregnancy, miscarriage, and live birth rates were not statistically different between women with and without HI and IR.

**Table 4:** Ovarian stimulation outcomes of women with hyperinsulinemia and insulin resistance.

	**Hyperinsulinemia**			**Insulin resistance**		
	**With *N* = 108**	**Without *N* = 996**	***P*-value**	**With *N* = 133**	**Without *N* = 971**	***P*-value**
Duration of stimulation (day)	**10.7** **±** **2.4**	**9.9** **±** **2.1**	**0.000**	**10.6** **±** **2.3**	**9.9** **±** **2.1**	**0.002**
Total gonadotropin dose (IU)	**2,409.5** **±** **812.4**	**2,171.4** **±** **860.8**	**0.005**	**2,353.6** **±** **775.3**	**2,173.0** **±** **867.6**	**0.014**
Peak E2 (pg/ml)	**2,149.1** **±** **1098.5**	**2,489.9** **±** **1,211.9**	**0.004**	**2,257.6** **±** **1,113.2**	**2,484.2** **±** **1,215.2**	**0.035**
Endometrial thickness (mm)	11.2 ± 2.7	10.8 ± 2.4	0.071	11.3 ± 2.6	10.8 ± 2.3	0.090
Number of oocytes retrieved	9.5 ± 4.2	9.4 ± 4.5	0.799	9.7 ± 4.3	9.4 ± 4.5	0.402
Number of 2PN	5.0 ± 2.8	5.0 ± 3.3	0.950	5.4 ± 3.1	5.0 ± 3.2	0.177
Clinical pregnancy (*n*, %)^a^	50/108 (46.2%)	392/996 (39.4%)	0.161	58/133 (43.6%)	382/971 (39.3%)	0.121
Live birth (*n*, %)^a^	30/108 (28.7%)	260/996 (23.3%)	0.732	40/133 (27.8%)	250/971 (23.2%)	0.681
Miscarriage (*n*, %)^b^	15/61 (24.6%)	137/491 (27.9%)	0.670	18/77 (23.3%)	134/475 (28.2%)	0.678

*E2, estradiol; 2PN, two-pronuclear embryo*.

a *Clinical pregnancy and live birth rates were calculated among all women*.

b *Miscarriage rate was calculated among pregnant women. Bold values indicate P-value < 0.05*.

### Linear Regression

FIN and HOMA-IR were viewed as continuous variables in linear regression to further explore the association with HI and IR. FIN was positively associated with T, FG, and TG levels, while negatively associated with P, LH, FSH, E2, and HDL levels. HOMA-IR was positively associated with T, FG, LDL, and TG levels, while negatively associated with P, LH, FSH, E2, and HDL levels. After adjusting for age and BMI, these associations still existed (Supplementary Table 1). For ovarian stimulation and reproductive outcomes, FIN and HOMA-IR were both positively associated with stimulation duration and total gonadotropin dose and negatively associated with peak E2. These associations were still significant after adjustment for age and BMI (Supplementary Table 2).

### Logistic Regression

Logistic regression showed that HI and IR were not associated with clinical pregnancy, live birth, and miscarriage (Table 5). Furthermore, when viewed as a continuous variable, FIN and HOMA-IR did not have associations with clinical pregnancy, live birth, and miscarriage (Supplementary Table 3).

**Table 5:** Reproductive outcomes in women with hyperinsulinemia and insulin resistance.

	**Hyperinsulinemia**	**Insulin resistance**
	Adjusted odds ratio^*^	Adjusted odds ratio^*^
	**(95% CI)**	**(95% CI)**
Clinical pregnancy^a^	1.34 (0.90–1.99)	1.44 (0.99–2.07)
Live birth^a^	1.33 (0.86–2.08)	1.28 (0.85–1.92)
Miscarriage^b^	0.86 (0.51–1.46)	0.97 (0.60–1.58)

a *Clinical pregnancy and live birth rates were calculated among all women*.

b *Miscarriage rate was calculated among pregnant women*.

* *Adjusted for age, BMI*.

## Discussion

In the current retrospective cohort study of women without PCOS who underwent assisted reproduction, HI and IR were associated with worse sex hormone and metabolic parameters. In addition, HI and IR were also associated with poorer cycle stimulation characteristics, including higher gonadotropin requirements, longer duration of gonadotropin stimulation, and lower peak E2 level. However, HI and IR did not affect reproductive outcomes including pregnancy, live birth, and miscarriage.

As expected, HI and IR were associated with metabolic hormones and sex hormones, which is similar in women with PCOS (15). HI and IR are characterized by abnormal secretion of sex hormones and insulin response. IR and HI can promote the liver secretion of very-low-density lipoproteins and activate liver endothelial lipase that promote HDL decomposition and therefore decrease HDL (21). Insulin inhibits the activation of TGlipase (a key rate-limiting enzyme for lipid mobilization), resulting in increased lipid mobilization and TG synthesis (22). HI and IR are also associated with the function of the hypothalamic-pituitary-ovary axis. Mice with conditional knockout of the insulin receptor in neurons show impaired maturation of the ovarian follicle due to hypothalamic dysregulation of LH (23). We did not find an association between HI, IR, and testosterone, but previous studies suggested that insulin is associated with concentrations of sex hormone-binding globulins, which might affect bioavailability of androgens (24).

In PCOS, enlarged ovarian volume and excess follicles were associated with IR (25). In women without PCOS, the total follicle count was significantly higher in those with a HOMA-IR >2.5, and HOMA-IR was positively correlated with the total follicle count in this group of women (26). IR was a predictor of ovarian hyperstimulation syndrome in women with non-PCOS (27). Our results showed that HI and IR were associated with a longer duration of ovarian stimulation, a higher total gonadotropin dose, and a lower peak E2 level, but not with the number of retrieved oocytes. Together, these findings suggested that HI and IR might inhibit folliculogenesis by suppressing FSH and increasing resistance to gonadotropin in follicle development (28). Although we did not find an association between HI, IR, and endometrial thickness, increasing the dose of gonadotropin and a longer duration of stimulation might indicate impaired endometrial receptivity.

HI and IR lead to poor reproductive outcomes in women with PCOS undergoing ovulation induction and assisted reproduction (15, 17). This could partly explain why drugs for controlling blood glucose could possibly improve reproductive outcomes in women with PCOS (29, 30). However, the current study found that HI and IR were not associated with clinical pregnancy rate, live birth rate, and miscarriage rate in women without PCOS. Although some studies reported that metabolic and ovarian stimulation outcomes might negatively affect reproductive outcomes (31–38), the current study did not recommend treating HI and IR for non-PCOS women undergoing assisted reproduction. However, the women in our study were relatively young, lean, and had a lower prevalence of HI and IR. Therefore, these associations still need research in different populations with a higher BMI.

Some previous studies have investigated clinical predictors of the starting dose of recombinant FSH in the assisted reproduction procedure. For example, a study found that obese women require significantly higher amounts of gonadotropins to achieve similar success rates in IVF compared to normal weight women (39). Another study used age and ovarian reserve markers to optimize the initial dose of recombinant FSH, leading to a more customized initial dose and improved cost-effectiveness. But for women with high AMH, it does not appear adequate (40). Our results indicated that IR and HI might help construct a better algorithm to decide the starting dose of controlled ovarian stimulation.

HI and IR are routinely monitored in clinical settings to assess diabetes and cardiovascular diseases. Considering the key role of IR in women with PCOS, insulin sensitizers, including metformin and inositol isoforms, can benefit women with PCOS due to their safety profile and effectiveness (41–43). With the modern obesity epidemic [44], the prevalence of HI and IR is believed to be increasing, even in women without PCOS. The current study suggests that there may be benefits in evaluating HI and IR status for young, lean women without PCOS before assisted reproduction treatment. Future clinical studies should focus on whether pretreatment improving HI and IR status is beneficial for women without PCOS.

The main strength of the current study was the large sample size. The observed associations remained significant after adjustment for age and BMI. But it also had several limitations. One is the design of the retrospective study. Although the large sample size, the adjustments made, and the multivariate regression analysis conducted, the presence of confounding bias cannot be completely excluded. Many women were excluded from the whole cohort due to the lack of glucose and insulin measurements, which could decrease the generalizability of our conclusion. Additionally, our population studied was relatively young and lean; more research is needed in specific subpopulations of patients with more frequent HI and IR, such as more obese women. Finally, as only a single measure of preconception serum insulin and glucose levels was available, it was impossible to investigate associations between HI and IR in sequential cycles and the cumulative live birth rate.

The results of the current study suggested that HI and IR impaired endocrine, metabolic, and ovarian stimulation outcomes but not reproductive outcomes in non-PCOS women undergoing assisted reproduction. However, more prospective studies are needed to confirm these associations.

## Data Availability Statement

The original contributions presented in the study are included in the article/Supplementary Material, further inquiries can be directed to the corresponding author/s.

## Ethics Statement

The studies involving human participants were reviewed and approved by Ethics Committee of the Women's Hospital, School of Medicine, Zhejiang University. The ethics committee waived the requirement of written informed consent for participation.

## Author Contributions

JX designed the study and critically revised the manuscript. W-YC and XL performed data analysis and drafted the manuscript. W-YC, XL, JS, CD, and WW collected data. DJ and JZ revised the manuscript. All authors contributed to the article and approved the submitted version.

## Conflict of Interest

The authors declare that the research was conducted in the absence of any commercial or financial relationships that could be construed as a potential conflict of interest.

## Publisher's Note

All claims expressed in this article are solely those of the authors and do not necessarily represent those of their affiliated organizations, or those of the publisher, the editors and the reviewers. Any product that may be evaluated in this article, or claim that may be made by its manufacturer, is not guaranteed or endorsed by the publisher.
