# Supplementary material for: Effect of Hyperinsulinemia and Insulin Resistance on Endocrine, Metabolic, and Reproductive Outcomes in Non-PCOS Women Undergoing Assisted Reproduction: A Retrospective Cohort Study
Source: Front Med (Lausanne). 2022 Jan 7;8:736320. doi: 10.3389/fmed.2021.736320 (PMC8777269; doi:10.3389/fmed.2021.736320)
Supplement: Supplementary file 1 [file Table_1.DOCX]

| **Supplemental table 1 Correlations between characteristics and FIN, HOMA-IR** | | | | |
| --- | --- | --- | --- | --- |
|  | FIN | | HOMA-IR | |
|  | β | Adjusted β* | β | Adjusted β* |
| Age (year) | 0.035 (-0.033 to 0.103) |  | 0.141 (-0.139 to 0.421) |  |
| Height (cm) | 0.007 (-0.129 to 0.143) | -0.008 (-0.140 to 0.123) | 0.057 (-0.500 to 0.614) | 0.007 (-0.532 to 0.547) |
| Weight (kg) | 0.097 (-0.030 to 0.224) | 0.023 (-0.033 to 0.078) | 0.363 (-0.157 to 0.883) | 0.110 (-0.117 to 0.338) |
| BMI (kg/m2) | 0.030 (-0.016 to 0.075) |  | 0.102 (-0.085 to 0.289) |  |
| Duration of infertility | 0.004 (-0.041 to 0.050) | 0.006 (-0.039 to 0.052) | 0.070 (-0.116 to 0.256) | 0.078 (-0.108 to 0.264) |
| AMH (ng/ml) | -0.015 (-0.033 to 0.103) | -0.017 (-0.049 to 0.016) | -0.059 (-0.192 to 0.073) | -0.063 (-0.196 to 0.069) |
| Progesterone (nmo/L) | **-0.020 (-0.032 to -0.008)** | **-0.020 (-0.032 to -0.008)** | **-0.079 (-0.127 to -0.030)** | **-0.079 (-0.127 to -0.031)** |
| Testosterone (nmol/L) | **0.012 (0.003 to 0.021)** | **0.012 (0.003 to 0.021)** | **0.050 (0.013 to 0.087)** | **0.049 (0.012 to 0.086)** |
| LH (IU/L) | **-0.089 (-0.131 to -0.048)** | **-0.090 (-0.132 to -0.048)** | **-0.381 (-0.551 to -0.221)** | **-0.384 (-0.554 to -0.214)** |
| FSH (IU/L) | **-0.098 (-0.140 to -0.055)** | **-0.098 (-0.140 to -0.055)** | **-0.392 (-0.566 to -0.218)** | **-0.391 (-0.565 to -0.218)** |
| E2 (pg/ml) | **-0.421 (-0.701 to -0.141)** | **-0.434 (-0.714 to -0.154)** | **-1.869 (-3.014 to -0.723)** | **-1.914(-3.059 to -0.770)** |
| Prolactin (ng/ml) | -0.083 (-0.292 to 0.125) | -0.087 (-0.296 to 0.121) | -0.273 (-1.118 to 0.572) | -0.286 (-1.132 to 0.559) |
| SBP (mmHg) | 0.064 (-0.109 to 0.237) | 0.052 (-0.120 to 0.224) | 0.222 (-0.485 to 0.929) | 0.177 (-0.526 to 0.880) |
| DBP (mmHg) | 0.127 (-0.015 to 0.269) | 0.114 (-0.026 to 0.254) | 0.429 (-0.152 to 1.010) | 0.381 (-0.192 to 0.955) |
| FG (mmol/L) | **0.029 (0.021 to 0.036)** | **0.029 (0.021 to 0.037)** | **0.203 (0.174 to 0.233)** | **0.204 (0.174 to 0.233)** |
| FIN (µU/ml) | **-** | **-** | **4.028 (3.984 to 4.071)** | **4.027 (3.983 to 4.070)** |
| HOMA-IR | **0.240 (0.238 to 0.243)** | **0.240 (0.238 to 0.243)** | **-** | **-** |
| HDL (mmol/L) | **-0.022 (-0.026 to -0.017)** | **-0.022 (-0.026 to -0.017)** | **-0.090 (-0.108 to -0.071)** | **-0.090 (-0.108 to -0.071)** |
| LDL (mmol/L) | 0.010 (-0.002 to 0.021) | 0.010 (-0.002 to 0.021) | **0.057 (0.010 to 0.104)** | **0.056 (0.009 to 0.104)** |
| TC (mmol/L) | -0.003 (-0.017 to 0.010) | -0.004 (-0.017 to 0.009) | 0.001 (-0.053 to 0.055) | 0.001 (-0.054 to 0.054) |
| TG (mmol/L) | **0.046 (0.037 to 0.055)** | **0.046 (0.037 to 0.055)** | **0.196 (0.159 to 0.232)** | **0.195 (0.158 to 0.232)** |
| BMI, body mass index; AMH, anti-Mullerian hormone; LH, luteinizing hormone; FSH, follicle-stimulating hormone; E2, estradiol; SBP, systolic blood pressure; DBP, diastolic blood pressure; FG, fasting glucose; FIN, fasting insulin; HOMA-IR, homeostatic model assessment-insulin resistance; HDL, high-density lipoprotein; LDL, low-density lipoprotein; TC, total cholesterol; TG, triglycerides  *Adjusted for age and body mass index  Bold values indicate P value < 0.05 | | | | |

| **Supplemental table 2 Correlations between ovarian stimulation outcomes and FIN, HOMA-IR** | | | | |
| --- | --- | --- | --- | --- |
|  | FIN | | HOMA-IR | |
|  | β | Adjusted β* | β | Adjusted β* |
| Duration of stimulation (day) | **0.067 (0.032 to 0.102)** | **0.066 (0.031 to 0.101)** | **0.264 (0.121 to 0.408)** | **0.261 (0.117 to 0.404)** |
| Total gonadotropin dose (IU) | **18.673 (4.721 to 32.625)** | **18.582 (4.607 to 32.557)** | **74.822 (17.689 to 131.954)** | **74.564 (17.350 to 131.777)** |
| Peak E2 (pg/ml) | **-26.400 (-46.375 to -6.425)** | **-26.533 (-46.550 to -6.515)** | **-123.375 (-205.134 to -41.616)** | **-123.854 (-205.765 to -41.942)** |
| Endometrial thickness (mm) | 0.030 (-0.009 to 0.069) | 0.030 (-0.010 to 0.069) | 0.106 (-0.056 to 0.268) | 0.105 (-0.057 to 0.267) |
| Number of oocytes retrieved | 0.024 (-0.048 to 0.096) | 0.021 (-0.051 to 0.094) | 0.055 (-0.241 to 0.352) | 0.045 (-0.251 to 0.341) |
| Number of 2PN | 0.004 (-0.049 to 0.056) | 0.003 (-0.049 to 0.056) | 0.010 (-0.205 to 0.225) | 0.010 (-0.206 to 0.225) |
| E2, estradiol; 2PN, 2 pronuclei; FIN, fasting insulin; HOMA-IR, homeostatic model assessment-insulin resistance  *Adjusted for age and body mass index  Bold values indicate P value < 0.05 | | | | |

| **Supplemental table 3 Association between reproductive outcomes with FIN and HOMA-IR** | | |
| --- | --- | --- |
|  | FIN | HOMA-IR |
|  | Adjusted OR (95%CI) * | Adjusted OR (95%CI) * |
| Clinical pregnancy ^a^ | 1.02 (0.99-1.05) | 1.08 (0.94-1.23) |
| Live birth ^a^ | 1.03 (0.99-1.07) | 1.11 (0.96-1.29) |
| Miscarriage ^b^ | 0.98 (0.94-1.03) | 0.93 (0.78-1.11) |
| FIN, fasting insulin; HOMA-IR, homeostatic model assessment-insulin resistance  ^a^ Clinical pregnancy and live birth rates were calculated among all women.  ^b^ Miscarriage rate was calculated among pregnant women.  * Adjusted for age, BMI | | |
